# Neratinib and the Role of Anti‐HER2 Therapy in Salivary Duct Carcinoma

**DOI:** 10.1002/cnr2.70065

**Published:** 2025-01-15

**Authors:** Martina Napolitano, Lucia Trudu, Enrica Martinelli, Chiara Santini, Massimo Dominici, Federica Bertolini

**Affiliations:** ^1^ Department of Oncology and Hematology Azienda Ospedaliero‐Universitaria di Modena Modena Italy; ^2^ Department of Medical and Surgical Sciences University of Modena and Reggio Emilia Modena Italy; ^3^ PhD Program Clinical and Experimental Medicine, Department of Biomedical Metabolic and Neural Sciences, University of Modena and Reggio Emilia Modena Italy; ^4^ AULSS7 Pedemontana—Unità Operativa Complessa di Oncologia Italy

**Keywords:** head and neck, HER2 targeted therapy, neratinib, precision medicine, salivary duct carcinoma

## Abstract

**Backgroud:**

Salivary duct carcinoma (SDC) is a rare and aggressive malignancy with a generally dismal prognosis and no standard of care established, despite a known association with epidermal growth factor receptor 2 (HER2) and androgen receptor (AR) over‐expression.

**Case:**

We report the case of a 64‐year‐old female with extra‐ and intracranial metastases of SDC with evidence of AR and HER2 overexpression. After progression on first line chemotherapy, was administered neratinib, a pan‐Erb2 receptor tyrosine kinase inhibitor.

Even with central nervous system involvement at diagnosis, a durable clinical response was obtained with a PFS of 11 months and no significant toxicities to manage. Best response observed during tratment was partial response.

**Conclusions:**

This case confirms the potential efficacy of neratinib in HER2‐positive SDC and underlines the need to define the best therapeutic sequence and potential biomarkers for these rare patients.

## 1 Introduction

Salivary duct carcinoma (SDC) is a rare and aggressive subtype of salivary gland cancers, sharing histological and molecular characteristics with ductal carcinoma of the breast [1, 17]. The prognosis of SDC is generally poor, largely due to its high rate of distant metastases, most commonly to the lungs and bones, with brain metastases also occurring relatively frequently [2, 3].

The majority of SDC cases (67%–97%) express the androgen receptor (AR), and approximately 45% exhibit overexpression of human epidermal growth factor receptor 2 (HER2) [4], making AR and HER2 key therapeutic targets in SDC. Both androgen deprivation therapy (ADT) and HER2‐targeted therapy have demonstrated clinical efficacy in metastatic SDC patients, as evidenced by Phase II clinical studies [5, 6]. However, ASCO guidelines emphasize the absence of evidence favoring any particular single‐agent or combination therapy, as no randomized Phase III trials have been published to date [7].

Neratinib, an oral, irreversible inhibitor of epidermal growth factor receptors (ErbB1/EGFR), HER2 (ErbB2), and HER4 (ErbB4), works by inhibiting tyrosine kinases phosphorylation and subsequent downstream signaling pathways [8].

Its approval by the FDA in the adjuvant setting for the extended treatment of early‐stage HER2‐positive breast cancer following trastuzumab therapy highlights its therapeutic potential [10]. Preclinical studies have demonstrated neratinib's ability to penetrate the brain, indicating potential central nervous system (CNS) activity [9]. Ongoing clinical trials are investigating neratinib both as a monotherapy and in combination regimens for HER2‐positive breast cancer with brain metastases, as well as for other malignancies such as HER2‐mutant non‐small cell lung cancer [10, 11, 12, 13].

Given its demonstrated efficacy in breast cancer, neratinib is expected to be effective in other HER2‐expressing tumors, including SDC.

Here, we present an emblematic case of HER2‐positive SDC with brain metastases treated with neratinib, resulting in clinical benefit, including an unexpected CNS radiological response.

## 2 Case Presentation

**FIGURE 1:**
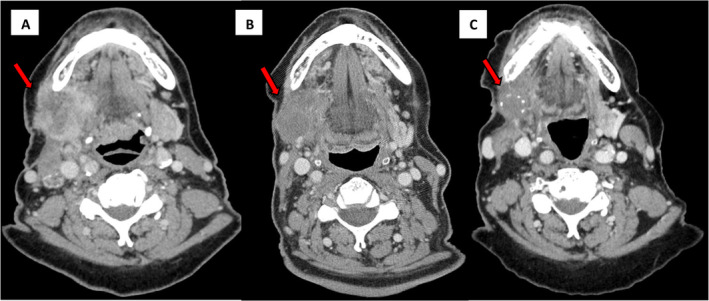
Head and neck computed tomography images. (A) CT scan revealed a solid mass of the right submandibular gland, infiltrating the right parotid gland and lower jaw, and bilateral cervical lymphadenopathy at baseline. (B) CT reexamination showed that both submandibular solid mass and cervical bilateral lymphadenopathy were significantly reduced after chemotherapy. (C) CT scan showed additional reduction of submandibular mass and cervical bilateral lymphadenopathy after 3 months of neratinib treatment.

**FIGURE 2:**
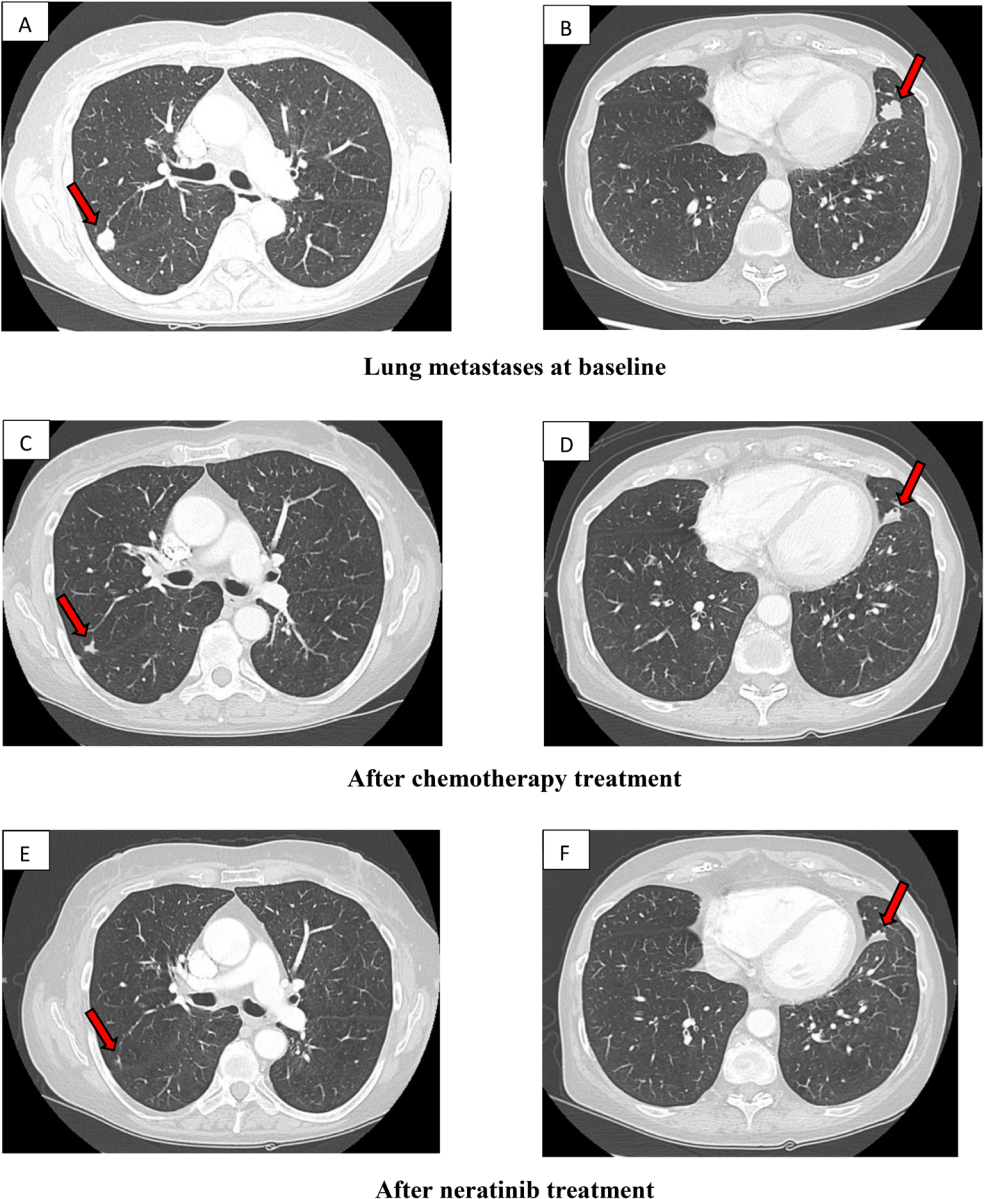
Chest computed tomography images. (A, B) CT scan revealed multiple lung metastases at baseline. (C, D) CT reexamination showed size reduction of lung metastases after chemotherapy treatment. (E, F) CT reexamination showed substantial stability of lung metastases after 3 months of neratinib treatment.

In December 2018, a 64‐year‐old female presented with pain and progressive right‐sided facial swelling. A total‐body computed tomography (CT) scan revealed a solid mass in the right submandibular gland, infiltrating the right parotid gland and lower jaw, along with bilateral cervical lymphadenopathy and lung metastases (Figure 1A; Figure 2A,B). A biopsy of submandibular mass confirmed a diagnosis of high‐grade SDC. Immunohistochemical analysis demonstrated overexpression of HER2 (3+) and AR (90%). Given the strong immunohistochemistry positivity, fluorescence in situ hybridization (FISH) analysis for HER2 was not performed. Due to the patient's claustrophobia and refusal, a positron emission tomography‐computed tomography (PET‐CT) scan was not carried out. The initial clinical stage was stage IVC (cT4a, cN3b, cM1) according to *American Joint Committee on Cancer* (AJCC) 8th edition.

At the time, antiHER2 plus chemotherapy regimens and first‐line ADT, were not yet approved in Italy for this type of tumor. Therefore, from March 2019 to July 2019, the patient received first line chemotherapy with cisplatin 60 mg/sqm and doxorubicin 50 mg/sqm, for 4 cycles, resulting in a partial response across all disease sites (Figure 1B; Figure 2C,D). The patient was then placed under surveillance, with periodic clinical and radiological evaluations.

Ten months later, the patient experienced increasing pain in the submandibular region. A CT‐scan revealed disease progession locally, in the lungs, and a suspected left frontal lobe brain metastasis. Brain magnetic resonance imaging (MRI) confirmed the left frontal lobe lesion and identified additional bilateral cerebral and cerebellar metastases. From July 2020 through September 2020, the patient underwent whole‐brain radiotherapy (WBRT) (30 Gy in 10 fractions) and palliative radiotherapy for pain control on the submandibular swelling (45 Gy in 15 fractions).

Next‐generation DNA sequencing (NGS) was performed on the diagnostic biopsy for further molecular characterization revealing no genetic alterations except a known amplification of the ERB‐B2 gene.

One month after completing radiotherapy, based on patient's clinical condition and molecular/histopathological findings she initiated neratinib (240 mg once daily) as part of compassionate use program. Prophylactic loperamide was administered to manage potential side effects. The treatment was well tolerated, with no adverse events, and the patient experienced notable clinical benefits, including effective pain control. Follow‐up imaging after 1 month showed a partial local response, with stable disease in the lungs and brain. Consequently, neratinib treatment was continued. In January 2021, the patient was hospitalized for acute exacerbation of chronic obstructive pulmonary disease (COPD) and tested positive for SARS‐CoV‐2. A CT scan at that time showed stable disease (Figure 1C; Figure 2E,F), and neratinib was resumed. Six months later, CT imaging confirmed stable disease in the lungs and locally, but a new osteolytic metastasis was identified at the fourth lumbar vertebra (L4). After multidisciplinary discussion, it was decided to continue neratinib and treat the L4 lesion with stereotactic radiotherapy (SBRT) (27 Gy in 3 fractions).

Three months later, during a subsequent restaging CT scan, progression was observed in lung nodules and mediastinal lymph node metastases, along with a new left anterior frontal brain metastasis. Given systemic disease progression and overexpression of AR (90%) in the biopsy tissue, ADT was proposed. Considering the patient's suboptimal health and her unfitness for chemotherapy combined with trastuzumab, ADT was initiated. In October 2021, the patient started bicalutamide (50 mg QD) and triptorelin. This regimen was maintained for 6 months until the patient experienced worsening fatigue, pain, and further decline in performance status. A subsequent CT scan confirmed progressive intracranial disease, and patient passed away 1 month later.

## 3 Discussion

This case highlights the potential of personalized therapies based on actionable genetic alterations as a promising approach to more individualized treatment strategies for managing rare cancers.

In cases of unresectable or metastatic SDC, the selection of systemic therapy should be tailored to individual patient factors, such as performance status and therapeutic goals [7, 14].

Systemic therapy for these patients is rapidly evolving, with targeted therapies emerging as a key option [15]. A significant proportion of advanced SDC are positive for AR and/or HER2 [16, 17], making it essential to assess both markers in all SDC patients [7, 14]. Strong evidence supports the use of anti‐HER2 targeted agents for HER2‐positive SDC as HER2 plays a central role in activating key signaling pathways, including the PI3K/AKT/mTOR and RAS/RAF/MEK/ERK cascades [18, 19].

Various TKI that interrupt this signaling pathways are now available, targeting different levels of receptor activation. Most commonly, monoclonal antibodies such as trastuzumab, pertuzumab, and TDM‐1 bind to extracellular epitopes of the HER2 receptor, while newer small molecule TKIs (smTKIs), including lapatinib, pyrotinib, neratinib, and tucatinib, inhibit downstream signaling more effectively [15]. Some of these smTKIs also demonstrate the ability to penetrate the blood–brain barrier, offering hope for treating CNS metastases [8, 20, 21].

Laurie et al. [22] and Takahashi et al. [6] recommended trastuzumab combined with chemotherapy as preferred first‐line approach in case of HER2 positivity for SDC. In this Phase II study conducted in Japan with 57 patients diagnosed with recurrent or metastatic HER2‐positive SDC, the combination of trastuzumab and docetaxel achieved a promising overall response rate (ORR) of 70.2%, including 14% complete responses and 56.1% partial responses. The median PFS was 8.9 months and OS was 39.7 months [6]. Similarly, a non‐randomized multi‐cohort basket trial investigating trastuzumab combined with pertuzumab in advanced HER2‐positive salivary gland cancers showed an ORR of 60%. Interestingly, among 5 patients with SDC (four of which were partial response) the ORR was 80% [23].

Ado‐trastuzumab emtansine (T‐DM1) has shown clinical efficacy in HER2‐positive SDC, as evidenced by case reports demonstrating responses in patients previously treated with other therapies [24, 25, 26]. Preliminary findings from a recent Phase II basket trial also support the use of T‐DM1 for patients whose disease progressed after trastuzumab therapy [27]. Recent research has highlighted the potential benefits of adjuvant trastuzumab in HER2‐positive SDC [28], and a prospective study evaluating adjuvant T‐DM1 is currently underway (NCT04620187). Furthermore, trastuzumab deruxtecan (T‐Dxd), another antibody‐drug conjugate (ADC) targeting HER2, has shown clinical benefit in HER2‐positive SDC patients in a Phase I basket trial [29]. Its effectiveness was also analyzed in a pooled analysis of two studies involving 17 patients with HER2‐positive SDC, in which this ADC demonstrated an ORR of 47%, with all responses being partial [30].

These studies suggest that monoclonal anti‐HER2 therapies can be effective for treating HER2‐positive SDC. However, there are significant limitations to these findings, including small patient populations, the inclusion of diverse and heterogeneous groups due to the rarity of the disease, and the lack of control groups in these single‐arm studies. Consequently, no monoclonal anti‐HER2 therapy has yet been standardized for the treatment of HER2‐positive SDC in clinical practice.

Neratinib, an irreversible pan‐HER receptor tyrosine kinase inhibitor (TKI), has demonstrated efficacy in HER2‐positive breast cancer and holds promise for SDC [9, 10, 11]. The ExteNET trial, a multicenter, randomized Phase III study, evaluated the efficacy of neratinib in patients with early‐stage HER2‐positive breast cancer, showing improved 2‐year invasive disease‐free survival (DFS) compared to placebo after trastuzumab therapy in an adjuvant setting (93.9% vs. 91.6%, *p* = 0.0091) [10]. Based on these findings, the FDA approved neratinib in 2017 for adjuvant use in patients with HER2‐positive breast cancer who have completed 1 year of trastuzumab therapy.

In the metastatic setting, Phase II studies showed a median PFS of 22.3 weeks and an ORR of 24% with better outcomes for patients who had not received prior trastuzumab [31]. The Phase III NALA trial compared neratinib combined with capecitabine to lapatinib plus capecitabine in HER2‐positive metastatic breast cancer who had undergone at least two previous HER2‐directed treatments, demonstrating improved PFS and CNS‐specific outcomes in the neratinib group [32, 33].

Additionally, a small retrospective case series reported that patients with leptomeningeal metastases from HER2‐positive breast cancer experienced neurological benefits from the neratinib and capecitabine combination, with a median duration of neurological response of 6.5 months [34].

Our case illustrates a transient clinical benefit from neratinib in a patient with metastatic SDC who experienced disease progression after chemotherapy. The patient achieved 11 months of PFS, with the treatment being well tolerated and allowing for further treatment upon disease progression. To the best of our knowledge, only two previous reports describe the use of neratinib in HER2‐positive SDC. Sorenson et al. reported the case of refractory metastatic HER2‐positive SDC with progressive cerebellar disease who received neratinib, however without clinical and radiological benefit [35]. Shukla et al. describes a case of HER2‐positive metastatic parotid gland carcinoma who achieved a complete response to trastuzumab deruxtecan after progression on neratinib and T‐DM1 [36].

Limited data are available on the use of ADT in combination with smTKIs. Yang et al. described a case of patient with advanced SDC treated pyrotinib and bicalutamide as third‐line therapy, achieving a durable partial response for 10 months [37], suggesting that dual targeting of HER2 and AR may be a salvage treatment option in advanced SDC. In our case, the patient was not eligible for conventional HER2‐targeted monoclonal antibody therapies, such as trastuzumab, due to local prescription restrictions, representing a limitation of the treatment in this case. Additionally, molecular analyses were only performed on the initial diagnostic biopsy, and no biopsy was conducted at disease recurrence, which could be a limitation, as HER2 and AR expression can evolve over time, as observed in breast cancer [38, 39]. Therefore, reevaluating these markers at disease recurrence or progression, could offer valuable insights for tailoring treatment. The use of circulating tumor cells (CTCs) for this purpose may also be promising, especially when tissue biopsies are challenging [40].

Our patient co‐expressed HER2 and AR, but the optimal treatment strategy and sequencing for such cases remain unclear. ADT and HER2‐targeted therapy have not yet undergone direct comparison. However, indirect comparisons from various studies suggest higher response rates and OS for HER2‐targeted therapy in combination with chemotherapy, over ADT alone [5, 19]. This is particularly evident in patients with visceral metastases and/or rapidly progressive disease, where HER2‐based treatment combined with a taxane appears to be the preferable initial option [41]. The EORTC1206, a randomized controlled Phase II trial, has been designed to clear whether dual androgen blockade (bicalutamide + triptorelin) is superior to cytotoxic chemotherapy in all AR‐positive SDC regardless of HER2 status (NCT01969578). AR positivity, along with the absence of estrogen and progesterone receptors, are recognized as histologic hallmarks of SDC. Notably, only 30% of SDC cases are HER2 positive [42]. Moreover preclinical findings showed that enzalutamide inhibits the growth of HER2 breast cancer cells, suggesting that the activity of AR inhibition might be anticipated in HER2 tumors, even independently of HER2 inhibition [43]. In a recent extensive retrospective study aimed at understanding the influence of HER2‐targeted therapy or ADT on patients with SDC who are both HER2 and AR positive, findings revealed that targeted therapy was linked to improved survival when compared to conventional therapy. Moreover, in this large cohort for ADT use the ORR was lower and the PFS was shorter in patients with HER2 positive SDC [44]. Interesting, among HER2 positive/AR positive SDC although HER2 targeted therapy had better ORR and PFS, no difference in OS was found. Taken together, even if HER2 targeted therapy can be recommended as first line approach, ADT is suitable in older patients or those with poor ECOG PS or multiple comorbidities. It should be noted that ADT is approved and used in females as well, as it targets the tumor, regardless of the patient's gender.

Finally, resistance mechanisms to targeted therapies and predictive biomarkers for treatment response in SDC remain poorly understood [45, 46, 47]. In a recent study by Saigusa et al. an investigation about EZH2 and H3K27me3 protein expression, along with EZH2 Y646 activating mutations, was conducted in a large cohort of SDC patients. The focus was on elucidating their prognostic and predictive significance concerning AR‐ or HER2‐targeted therapy. The findings revealed that elevated levels of EZH2 and H3K27me3 in patients undergoing AR‐targeted therapy correlated with shorter overall survival. However, no significant association was observed between EZH2 and H3K27me3 expression and clinical outcomes in patients receiving conventional or HER2‐targeted therapy [48]. How to apply these findings in clinical practice remains challenging.

## 4 Conclusions

Historically, one of the primary challenges in managing SDC has been the limited availability of therapeutic options and the difficulty in determining optimal treatment sequences. Although recent advances have identified actionable targets such as AR and HER2, these challenges remain unresolved. Our case underscores the importance of considering molecular signatures, particularly those related to the HER2 and AR pathways, early in the diagnostic process for this rare tumor type. Looking ahead, the introduction of personalized therapies, including the use of conjugated or unconjugated anti‐HER2 monoclonal antibodies, TKIs, and potentially in combination with ADT, may offer new strategies to improve outcomes for patients with this aggressive malignancy.

## Author Contributions

**Martina Napolitano:** writing – original draft, writing – review and editing. **Lucia Trudu:** visualization, writing – original draft, writing – review and editing. **Enrica Martinelli:** writing – review and editing, visualization. **Chiara Santini:** writing – original draft. **Massimo Dominici:** writing – review and editing, supervision. **Federica Bertolini:** conceptualization, supervision.

## Consent

Written informed consent was obtained from the patient. The pharmaceutical company provided consent for the publication of this case.

## Conflicts of Interest

The authors declare no conflicts of interest.

## Data Availability

The data that support the findings of this study are not openly available.
